# Single Molecule Studies on Dynamics in Liquid Crystals

**DOI:** 10.3390/ijms141019506

**Published:** 2013-09-26

**Authors:** Daniela Täuber, Christian von Borczyskowski

**Affiliations:** Institute of Physics and nanoMA, Technische Universität Chemnitz, Chemnitz D-09107, Germany; E-Mail: borczyskowski@physik.tu-chemnitz.de

**Keywords:** structure and dynamics, surfaces and external fields, single molecule, liquid crystal, nematic, smectic, lyotropic, thermotropic, diffusion, polarization, conjugated polymer, biopolymer

## Abstract

Single molecule (SM) methods are able to resolve structure related dynamics of guest molecules in liquid crystals (LC). Highly diluted small dye molecules on the one hand explore structure formation and LC dynamics, on the other hand they report about a distortion caused by the guest molecules. The anisotropic structure of LC materials is used to retrieve specific conformation related properties of larger guest molecules like conjugated polymers. This in particular sheds light on organization mechanisms within biological cells, where large molecules are found in nematic LC surroundings. This review gives a short overview related to the application of highly sensitive SM detection schemes in LC.

## 1. Introduction

Liquid crystals (LC) had been discovered for the first time in cholesterol found in carrots by F. Reinitzer [1]. More than 100 years later, investigation of biological LC materials is still of interest [2–4]. However, the focus has shifted from the study of general LC features to more sophisticated subjects such as their impact on molecular organization relevant for biological processes [2–5]. Living cells contain liquid crystalline environments e.g., cell membranes and stabilized analogues of liquid crystals [2]. These environments one the one hand impose a specific molecular organization on other solvated molecules and aggregations [5]. On the other hand, the morphogenesis of the environments itself is of interest. For example, collagens, major macromolecules of biological tissues, are known to form liquid crystalline phases at high concentrations *in vitro* [4]. Although, their morphogenesis *in vivo* is guided by enzymes, the liquid crystalline order prior to fibril assembly obviously is related to the final outcome of structure formation [2,4]. Besides resolving biological processes, investigations of biological liquid crystal environments are related to biomimetics, as they are expected to contribute extended insight for tissue engineering [6].

The most perceived applications of liquid crystals are LC displays [7]. However, their electrooptical and electromechanical properties have led to further important applications, like organic solar cells [8,9] and light polarizing devices [9,10]. Electrocative liquid crystalline polymers also find applications as actuators and sensors [9,11]. Extensive research on new LC materials [12,13] as well as on LC structure formation, electrooptical properties [14] and interactions with other materials is aimed to improve and extend LC based devices and other applications [15,16].

Many LC properties have been found from ensemble methods, such as light polarization microscopy and nuclear magnetic resonance (NMR, see for example [17]). However, the power of ensemble methods to retrieve information on local structure and interactions is restricted in respect to heterogeneous materials and dynamics. Here, single molecule (SM) methods can fill the gap, because investigation of the dynamics of single fluorescent molecules provides insight into local interactions between the medium and the single chromophore [18,19]. This, on the one hand, yields information on the local structure of the environment. On the other hand, the anisotropic LC structure has been used to study specific properties of the guest molecules themselves [20]. The very low concentration of dye molecules used within SM methods avoids contributions form dye-dye interactions, which otherwise might be present in optical probe based ensemble studies due to the need for higher dye concentrations. Typical dye concentrations for SM studies are in the nanomolar to micromolar range. This renders nonlinear optical effects negligible which otherwise have been observed for dye concentrations of 1% in liquid crystal [21]. Since their implementation, SM methods have been used in a broad field of soft matter materials [18,19] including dynamics in ultrathin films [22–27] or nanopores [28,29] and on surfaces [30,31]. Corresponding investigations in the field of LC are rare.

Up to now, SM studies have contributed to the investigation of physical properties (diffusion and conformational properties of guest molecules, local structure) of several LC materials. Here we first concentrate on the investigation of LC cells containing the nematic mixture E7 (a blend of a cyanoterphenyl and three cyanobiphenyls [11]) [32,33], the nematic 4-n-pentyl-4-cyanobiphenyl (5CB), as well as the smectic A 4-n-octyl-4-cyanobiphenyl (8CB) [34] via small tracer molecules. Small tracers were also used to investigate structure and dynamics of thin frustrated 8CB films on silicon substrates [35,36]. These SM studies were conducted at room temperature without the employment of temperature controlled sample stages. The self-diffusion coefficients of the used LC-materials show a considerable temperature dependence [17]. Nevertheless, a tabular comparison including results from ensemble experiments is given.

The anisotropic alignment of nematic 5CB and nematic/smectic 8CB was further used to investigate interactions with the conjugated polymer poly(2-methoxy-5-(2′-ethyl-hexyloxy)-*p*-phenylene vinylene (MEH-PPV) [20]. On the one hand, this contributed information about ordering properties and anisotropic diffusion of MEH-PPV [20]. On the other hand, the particular alignment of MEH-PPV in 5CB was used to retrieve local LC ordering across a 12.7 μm thick 5CB cell with and without application of an electric field [37].

Single molecule methods have also been applied to biological LC materials, which will be the topic of the last section. Dogic *et al*. used single molecule tracking (SMT) to study the diffusion behavior of *fd* viruses in aqueous solution, a lyotropic nematic LC [38], as well as the behavior of large biopolymers therein [41].

## 2. Single Molecule Investigation of LC Cells Using Small Dye Molecules

To our knowledge, the first application of single molecule (SM) methods to liquid crystals was the investigation of structure related dynamics of small dye molecules in nematic LC cells by Kawai *et al*. [32], which will be described in the following together with a more recent study of SM in nematic and smectic LC cells. Experiments using large conjugate polymer molecules within LC cells will be described in a later section.

### 2.1. Diffusion Anisotropy of Small Solute Molecules from SM in Nematic LC Cells

Kawai *et al*. were the first to report SM studies on thermotropic liquid crystals. They investigated the diffusion of a fluorescent perylene dye in a parallel aligned nematic LC cell using the liquid crystal mixture E7 by employing fluorescence correlation spectroscopy (FCS) [32,33]. Without the external electric field, the LC director was aligned in the laboratory *x*-direction, which was confirmed by polarized microscopy. The cell thickness of about 1 μm matched the focal depth of the confocal beam. For this reason, a 2-dimensional (2D) correlation function could be used for retrieving diffusion coefficients from experimental autocorrelations of the fluorescence intensity. The in-plane radius *w*_0_ of the Gaussian focus was determined from the wavelength λ, the refractive index *n* and the numerical aperture *A_N_* of the objective using

(1) $$w_{0}=\frac{2\lambda }{\pi nA_{N}}$$

Because of the optical anisotropy of the LC with refractive index *n*_⊥_ = 1.52 perpendicular to the LC director (laboratory *y*-direction) and *n*_||_ = 1.75 parallel to the LC director (laboratory *x*-direction), different focal half-widths *w_x_* and *w_y_* were retrieved from Equation (1). The optical anisotropy led to a 2D-correlation function employing different terms for the *x*- and *y*-directions [32]

(2) $$G(\tau )=\frac{1}{N}\frac{1}{\sqrt{1+\frac{4D_{x}}{w_{x}^{2}}\tau }}\frac{1}{\sqrt{1+\frac{4D_{y}}{w_{y}^{2}}\tau }}+1$$

with *N* the average number of molecules in the focus, and diffusion coefficients *D_x_* (*D*_||_) and *D_y_* (*D*_⊥_) in *x*- and *y*-direction, respectively. Fitting autocorrelation curves obtained from the LC cell without external electric field led to average diffusion coefficients *D_x_* = 18 μm^2^/s and *D_y_* = 3.0 μm^2^/s. The average diffusion anisotropy was obtained as *D*_||_*=D*_⊥_ = 4, with a maximum value of 6.7, see also Table 1. The average fitting error was given as 20%. The obtained diffusion anisotropy is much larger as *D*_||_*=D*_⊥_ ≈1.2 from ensemble measurements employing fluorescence recovery after photobleaching (FRAP) [42] (see Table 1). One explanation of the results from the SM study was that this difference might be due to the structure of the employed perylene diimide, which has long alkyl chains, and therefore might enhance the anisotropy in the translational diffusion in the nematic LC.

For this reason, Kawai *et al*. in a second study concentrated on the possible influence from dye structure on the diffusion anisotropy [33]. The used three antraquinone derivates had similar chromophores but different alkyl chain lengths. All three dye molecules showed a similar diffusion anisotropy *D*_||_*=D*_⊥_ with averages of about 5 (see Table 1). The broad distribution of the anisotropy ranged from 1.7 to 100 for all three dyes. This shows that the diffusion anisotropy does not depend on the alkyl chain length. The value roughly coincides with the value obtained in the previous study employing a perylene diimide and the same E7 medium. The difference between the diffusion anisotropy obtained by fluorescence recovery and by FCS is suggested to result from averaging over a larger sample area and a longer measurement time in the former as compared to the latter [32,33]. This demonstrates the potential of the SM method to directly explore the local environment of dye tracer molecules.

Kawai *et al*. in their first study also determined diffusion coefficients *D_x_* and *D_y_* for the cell under application of an external electric field using Equation (2). The average values were 12.0 μm^2^/s and 10.5 μm^2^/s, respectively. The weak diffusion anisotropy *D*_||_*=D*_⊥_ ≈ 1.1 indicates an almost isotropic in-plane diffusion of the perylene diimide. Thereby, the interfacial aligned LC layers at the cell walls contribute to the anisotropy [32]. Further information about the local LC alignment close to the cell walls of a parallel cell under application of an E-field was obtained from a study employing the conjugated polymer MEH-PPV in a nematic 5CB cell [37], which will be reported later.

### 2.2. Locally Resolved Structure Related Dynamics in Nematic 5CB and Smectic 8CB Cells

Recently, Pumpa and Cichos used single molecule tracking (SMT) to study the diffusion of a perylene diimide in LC cells of the nematic compound 4-n-pentyl-4-cyanobiphenyl (5CB) and its room temperature smectic A homolog 4-n-octyl-4-cyanobiphenyl (8CB) [34]. The self-diffusion for both LC materials is reported from NMR studies in a wide temperature range, yielding anisotropy values up to 2.5 for 5CB and 1.7 for 8CB at room temperature [17]. Pumpa and Cichos analyzed their SMT data via orientationally resolved distributions of single diffusion steps at a fixed time interval given by the inverse frame rate of the measurement. By this, they derived angle dependent diffusion coefficients for different regions of the sample. The local LC director in these regions was evaluated by polarization contrast microscopy. A comparison showed agreement of the dynamical properties reported by the dye molecules to the structural properties of the liquid crystal [34].

**Figure 1:**
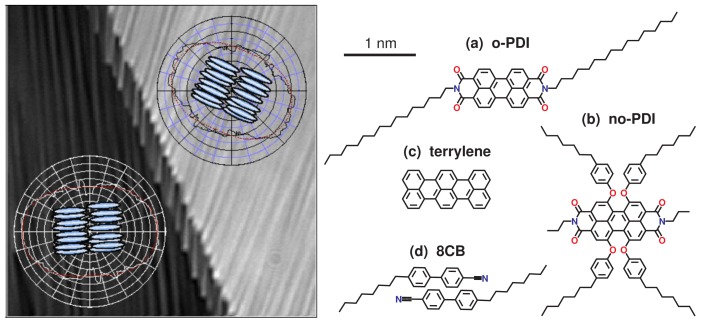
(**Left**) 8CB cell with two regions of ordering (bottom left) area 1, and (top right) area 2. Schematics of LC orientation are given together with the angular diffusion maps and their fits using a model for anisotropic diffusion (red lines) for each region. Reprinted with permission from [34], Copyright 2012 by The American Chemical Society. (**Right**) Molecular structure of (**a**) aligning perylene diimide o-PDI; (**b**) not aligning perylene diimide no-PDI; (**c**) terrylene; and (**d**) columnar LC unit of 8CB.

For nematic 5CB the diffusion coefficients were reported to be *D*_||_ = 8.47 μm^2^/s and *D*_⊥_ = 5.75 μm^2^/s, see also Table 1. For smectic 8CB, two regions (area 1 and area 2, see Figure 1 left) with different director alignment were studied, yielding comparable diffusion coefficients *D*_||_ = 4.5 ± 0.2 μm^2^/s and *D*_⊥_ = 2.9 ± 0.2 μm^2^/s for area 1, and *D*_||_ = 4.3 ± 0.2 μm^2^/s and *D*_⊥_ = 2.8 ± 0.2 μm^2^/s for area 2, which showed a slightly weaker director alignment than area 1.

Despite the good agreement of the anisotropy values, the absolute values of the diffusion tensors’ principle components deviate strongly from NMR data reported in literature [17], and also from diffusion coefficients obtained by forced Rayleigh scattering (FRS) using methyl red in 0.1 wt% concentration [43], see Table 1. According to the Stokes-Einstein relation, a small deviation is expected. Due to the slightly larger van der Waals radii of the tracer perylene molecules in comparison to the liquid crystal molecules, diffusion coefficients obtained by SMT should be smaller than those retrieved from the observation of self-diffusion by NMR. However, the observed deviation by a factor of about 6 cannot be explained merely by size difference. Pumpa and Cichos also conducted FRAP on their samples with a higher concentration of the perylene diimide. The average diffusion coefficients *D̄* (5CB) = 2.6 ± 0.8 μm^2^/s and *D̄* (8CB) = 3.2 ± 0.6 μm^2^/s are of similar range as the values obtained from SMT analysis [34], see also Table 1. A comparative FRAP experiment employing Rhodamine 6G in a glycerine/water mixture with a viscosity similar to the mean viscosity of 5CB, yielded a diffusion coefficient in good agreement with predictions from the Stokes-Einstein relation. This suggests that the observed slow molecular diffusion of perylene diimide in 5CB and 8CB must be related to the interaction of the dye molecule with the liquid crystal [34]. Investigations on hydrodynamics of spherical particles in nematic LC revealed larger effective viscosity due to necessary reorientation of LC director structure at the particles’ surface [44,45]. Similar slow diffusion coefficients have been reported by Schulz *et al*. using SMT of perylene diimides in thin frustrated smectic 8CB films [35,36], which will be described in the following.

## 3. SM Studies on Diffusion and Structure Related Dynamics in Thin Frustrated 8CB Films

Schulz *et al*. combined SMT and FCS of two different perylene diimides and terrylene (for molecular structures see Figure 1 right) to study structure related dynamics in thin frustrated smectic LC films of 8CB [35,36]. One of the perylene diimides (o-PDI) has been designed to align along the director of 8CB, the other one (no-PDI) did not show any alignment. Order parameters *S* for alignment in a 5CB cell were obtained as *S* = 0.7 and *S <* 0.1 for o-PDI and no-PDI, respectively [35]. Terrylene was used for comparison. Due to its comparatively small size, it should not disturb the LC structure to a large extent and probably also aligns along the director of 8CB. This study is aimed to investigate the influence of the interface and of self-organized structures on diffusion. Silicon substrates with either native or 100 nm thermal oxide were used with films of thickness *d* between 4 and 225 nm. The alignment of 8CB is known to be random planar at the silicon substrate [46] and homeotropic at the interface with air [47]. For the 4 nm thick 8CB film, the structure consists of a planar monolayer at the substrate covered by a bilayer of homeotropically aligned 8CB dimers [46,48]. For thicker films, the range and the kind of the distortions of the smectic layers close to the substrate are not fully understood [49].

**Figure 2:**
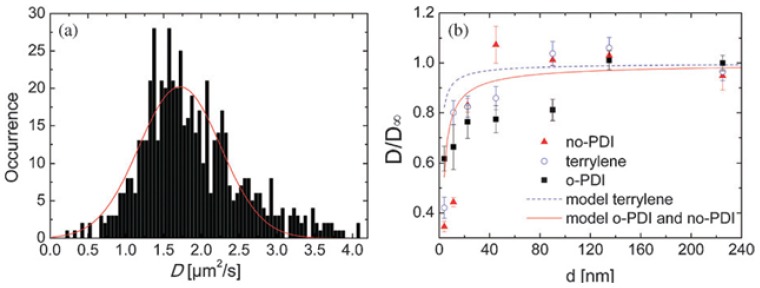
(**a**) Distribution of diffusion coefficients *D* calculated from trajectories of no-PDI within a 0.2 μm thick 8CB film on 100 nm SiO_2_. The full line shows a Gaussian fit to the distribution; (**b**) Normalized average diffusion coefficients as a function of film thickness *d* for 8CB on 100 nm SiO_2_ together with calculations using a hydrodynamic no-slip model. Image taken from [35], Copyright 2010 by The Royal Society of Chemistry, with permission.

Figure 2a shows the distribution of diffusion coefficients *D* obtained from trajectory analysis of SMT for no-PDI in a 0.2 μm thick 8CB film on 100 nm SiO_2_ together with a Gaussian fit for retrieving a mean diffusion coefficient. In general, by trajectory analysis of SMT distributions of diffusion coefficients are obtained, which is due to the experimentally limited observation time [22]. In case of the here studied tracer diffusion in 8CB films, the diffusion anisotropy leads to further broadening of the distribution. Schulz *et al*. additionally analyzed their SMT experiments via probability distributions of diffusivities, which are scaled square displacements of detected tracers at a fixed time time lang (scaled single steps) [26,35,36]. Within these probability distributions of diffusivities, deviations from homogeneous diffusion can be easily seen as deviations from a mono-exponential decay. For no-PDI in 0.2 μm thick 8CB films on 100 nm SiO_2_, multicomponent fits yielded two diffusion coefficients *D*_1_ = 2.4±0.3 μm^2^/s and *D*_2_ = 3.8 ± 0.6 μm^2^/s and a possible third component *D*_3_ = 105 ± 30 μm^2^/s [26], see Table 1. The values for *D*_1,2_ are similar to *D*_⊥_ = 2.8±0.2 μm^2^/s and *D*_||_ = 4.3±0.2 μm^2^/s obtained by Cichos *et al*. via SMT for a perylene diimide in a 0.5 μm thick 8CB cell.

Mean diffusion coefficients obtained from trajectory analysis of SMT using no-PDI and terrylene with varied 8CB film thickness showed a general agreement with a hydrodynamic model with no-slip (stick) boundary condition at the substrate [35], see Figure 2b. For the isotropically oriented dye no-PDI, the mean diffusion coefficient reached the plateau value (considered as bulk diffusion coefficient) already at *d* ≈ 40 nm. In contrast, for the aligning perylene diimide o-PDI a notable deviation from the hydrodynamic model was observed. The diffusion coefficients were significantly reduced up to 100 nm film thickness [35]. This deviation is suggested to be caused by the sensitivity of o-PDI to the structure change in the interface region in combination with the elongated shape of the molecule. A crossover from planar alignment at the substrate to the surface normal alignment of the smectic director might be accompanied by a decrease in the effective hydrodynamic radius of o-PDI [35]. An additional influence is expected from reversible adsorption events to the substrate along detected trajectories. Diffusivity analysis of the SMT experiments [26,35] showed a considerable adsorption probability for terrylene (72%) and o-PDI (62%) molecules in contrast to no-PDI (17%). Reversible adsorption to the surface along a dye trajectory will lead to an apparent slow down of diffusion coefficients from trajectory analysis. The slightly decreased mean diffusion coefficients for terrylene in respect to the hydrodynamic model for *d* up to 45 nm point to an influence from adsorption events up to this film thickness. This influence is reduced for the isotropic no-PDI, leading to a qualitative agreement of experimental results with the hydrodynamic model in case of no-PDI [35]. Recent atomistic molecular dynamics (MD) simulations for a 24 nm thick nematic 5CB film on amorphous silica showed a crossover from planar to substrate-normal alignment within 15 nm from the substrate [50]. For smectic 8CB the situation is more complex and thus MD simulations are still lacking. The above described results for the isotropic no-PDI suggest the crossover to occur within 40 nm from the substrate.

FCS measurements of o-PDI and no-PDI in 8CB films with varied thickness *d* also show decreasing correlation times (increasing diffusion coefficients) with increasing film thickness. For both dyes, “bulk” diffusion coefficients are reached for *d* ≥ 80 nm. The FCS curves had been fitted for either anomalous or two-component translational 2D-diffusion, because the usual one-component 2D function did not fit the experimental data. Diffusion coefficients from two-component fits to FCS curves for both perylene divides and terrylene in 0.2 μm thick 8CB films are given in Table 1, whereby an erroneous value in [35] for no-PDI is corrected. The ratio between the slow and fast components derived by the two-component 2D-fits ranges between two and three orders of magnitude. Kawai *et al*. had reported diffusion anisotropies ranging from 1.7 to 100 [33]. This points to local differences in diffusion anisotropy. However, the slow components from FCS were one order of magnitude slower than those obtained by SMT. This is quite unusual, since SMT in general is more sensitive to slow diffusion than FCS [26,51]. A further, not yet published study points to an influence of vertical translational diffusion on the FCS signal [52]. Due to reflections on the silicon substrate [53], the fluorescence intensity of vertically diffusing dye molecules strongly changes on a scale of ≈ 40 nm, which is smaller than most of the investigated film thicknesses *d* [52]. This probably will explain the apparent slower diffusion coefficients in respect to results from SMT, and thus also will reduce the obtained diffusion anisotropy. The faster component derived from the two-component fits matched the fastest component from diffusivity analysis of SMT and was suggested to result from fast LC domain fluctuations [36,54]. Maps of diffusivities from SMT showed a spatially inhomogeneous distribution of detected o-PDI molecules, whereas no-PDI was homogeneously distributed within the analyzed region [35]. The detection of o-PDI depends on the molecular orientation and this in turn follows the LC structure. The scale of the inhomogeneities is in the order of microns, which agrees with the size of 8CB domains observed on MoS_2_[55].

**Figure 3:**
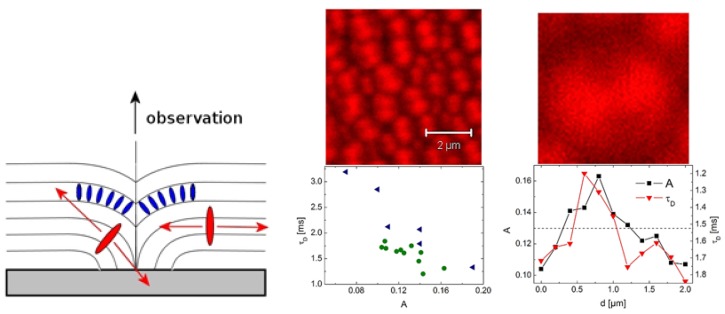
(**Left**) Schematic presentation of a focal conic domain (FCD) with incorporated o-PDI. Preferred directions of emission of o-PDI molecules in different areas of FCD are indicated by arrows. (**Middle** and **Right**) Correlation between the amplitude *A* of the autocorrelation function and the characteristic time *τ_D_* for o-PDI. Bottom middle: *τ_D_* as a function of the corresponding amplitude *A* for two independent measurements. The absolute values of *A* depend on the arbitrarily chosen scanning line across a typical sample (top middle). Bottom right: amplitude *A* and *τ_D_* as a function of the spatial position *d* along an arbitrarily chosen scan across the sample (through a FCD) with steps of length 200 nm. Lines are for eye guide. The broken line denotes half of the maximum intensity. On top right an image section of 2 microns size is shown for comparison. Note that the graphs on top show fluorescence emission from a different sample with higher dye concentration than the data shown below. Images and captions taken from [36], Copyright 2011 by The Royal Society of Chemistry, with permission.

Smectic-nematic-smectic temperature cycling of 225 nm thick 8CB films was performed to study the influence of local structure formation on molecular mobility [36]. Thin 8CB films on *Si* with native oxide are known to show surface textures at the smectic-nematic phase transition [56], which due to a memory effect may stay visible when cooled back into the smectic phase [57]. Schulz *et al*. observed an influence of the silicon oxide thickness upon structure formation, which points to contributions from long range van der Waals forces [26]. On 100 nm thermal oxide, focal conic domains (FCD) were observed, while in the film on native oxide, terraced holes within an otherwise flat film surface appeared [36]. Figure 3 (left) gives a schematic presentation of a FCD with incorporated o-PDI molecules. Tracer diffusion (FCS and SMT) before and after temperature cycling revealed a slight enhancement of diffusion after structure formation in case of no-PDI. The structure formation thus enhances the mobility of the isotropically oriented guest molecules in respect to the as-prepared film. For o-PDI, trajectory analysis of SMT yielded no change within experimental errors, while from fits to FCS curves, shorter correlation times were obtained [36], which are assigned to structure changes of the LC film. In contrast to no-PDI, o-PDI follows the LC director and thus can be used to map out the diffusion dynamics related directly to a single FCD. FCS measurements along a scan line employing o-PDI in an 8CB film containing FCD, revealed a correlated change in amplitude and correlation time along the scan line, which can be assigned to the structural change across a FCD [36], see Figure 3 (middle and right).

## 4. Conjugated Polymers in Liquid Crystal Matrices

One particular application of single molecule methods in liquid crystals is the investigation of the physical properties of conjugated polymers. Due to their high fluorescence and low cost, conjugated polymers are competing materials for the use as light emitting devices [58]. Since their fluorescence properties are closely related to conformations, liquid crystals were used as a host to study the interdependence of physical properties on conformations. Barbara *et al*. investigated the conjugated polymer poly(2-methoxy-5-(2′-ethyl-hexyloxy)-*p*-phenylene vinylene (MEH-PPV) in nematic and smectic liquid crystals employing single molecule methods [20,59–62]. A detailed summary is given in the review of their work by Barbara *et al*. [20]. A short summary of the results will be given in the following.

### 4.1. Order Parameters of MEH-PPV in Nematic 5CB

Order parameters of MEH-PPV in nematic 5CB were obtained from single-molecule polarization data in a confocal microscope equipped with two orthogonally polarized detection channels [59–62]. The LC cell was mounted on a rotation stage, allowing the LC director to be orientated at an angle with respect to the lab *y*-axis. It is worth to note that a modification of this “emission-selective” setup into an “excitation selective” one by using an unpolarized detection channel and two orthogonal linear polarized excitation beams chopped at a rate of 10 kHz yielded indistinguishable polarization distributions [60]. The intention for using the different setups was to show that the obtained polarization data were not distorted by energy transfer effects. However, Durbin *et al*. had reported on laser-induced optical reorientation taking place in 5CB already at a laser excitation of 150 W*=*cm^2^ when using linear polarized excitation oriented perpendicular to the LC director [63]. The “excitation selective” setup used by Link *et al*. employs linear polarized excitation also in the direction perpendicular to the LC director at a comparable intensity. A laser-induced tilt of the 5CB molecules should influence the obtained polarization of the MEH-PPV molecules. The observation of similar results to those employing circular polarized excitation shows that this was not the case. It is reasonable that the fast chopping rate of 10 kHz prevented the 5CB molecules from reorientation.

Individual polymer molecules diffusing through the excitation volume yield fluorescence bursts. The polarization ratio *P* was determined for each burst from the polarized florescence intensities in the individual channels and was accumulated into histograms. The molecules were observed to retain their characteristic polarization ratios throughout their diffusion times, suggesting that translation through the focal volume occurs with little or no rotational reorientation [59]. Measurements were employed for three different orientations (γ = 0, *π*/4, *π/*2) of the director with respect to the two detectors [59]. When the director is parallel to either polarization of one detector, the measured MEH-PPV bursts in the dual-channel intensity trajectories exhibit significant polarization bias in that particular direction. This behavior suggests that polymer molecules are both highly aligned along the director and highly polarized. With the director at 45° (γ = π/4), no polarization bias is evident in the intensity data, and the obtained polarization histogram is peaked at zero. From these data, the authors derived an orientational order parameter *S_o_* = 0.99 of MEH-PPV in 5CB. Since MEH-PPV chains consist of several segments, also a conformational order parameter *S_c_* = 0.51 was determined [60].

Both order parameters obtained from single molecule polarization are considerably larger than the solute order parameter *S* = 0.30 derived from ensemble experiments on MEH-PPV in 5CB [20]. The discrepancy may be a result of several complications with the ensemble experiment, including (a) a greater sensitivity to lower-molecular-weight chains, which may be less well aligned; (b) the scrambling effect of interchain energy transfer, which is more prevalent for the higher-concentration samples used in ensemble measurements; and (c) imperfections in the single-LC structure, which are a more serious complication for the larger sample areas used in the ensemble measurements [20]. The higher sensitivity of the single molecule experiment to longer, straighter, and thus better aligned chains (a) was confirmed by a further study on MEH-PPV conformation in 5CB [64]. A comparison of the conformational order parameters *S_c_* obtained from single molecule polarization measurements of MEH-PPV in 5CB and in a PMMA (poly(methyl methacrylate) polymer matrix reveals that the polymer chains are stretched out in an anisotropic solvent (5CB: *S_c_* = 0.50 – 0.60, PMMA: *S_c_* = 0.40) in agreement with predictions from Onsager [65]. From the observation of a higher-orientational order parameter for the solute than that of the solvent it can be concluded that the potential experienced by MEH-PPV comprises an additive and correlated contribution of tens to hundreds of solvent molecules. The potential of long-range solvation forces to produce an orientationally and conformationally ordered solute was further confirmed by investigation of MEH-PPV with an increased number of chain segments [60].

**Figure 4:**
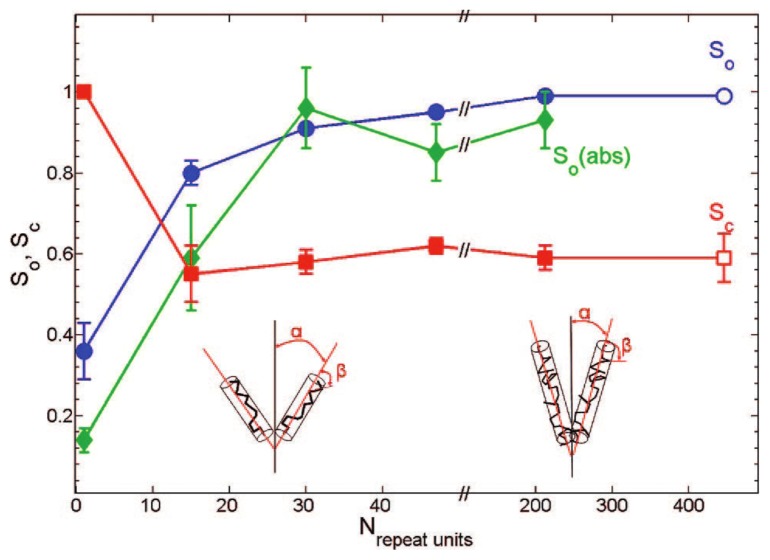
Orientation *S_o_* and conformation *S_c_* order parameters as a function of repeat units *N*. Results of an independently measured sample with 423 repeat units are included for comparison (open symbols). Green diamonds are data points from ensemble absorption measurements. Lines are given as guides to the eyes. The cartoon (not to scale) shows qualitatively how the ordering of a polymer in an LC is enhanced with increasing polymer chain length. Image and caption reprinted with permission from [64], Copyright 2008 by The American Chemical Society.

Tcherniak *et al*. used the above described single molecule polarization method [59–62] to study the dependence of the solute order parameter *S_o_* on the solute molecular weight *M* ranging from monomeric Rhodamine 6 G to 432 repeat units (*M* = 3900) [64]. While the solute order parameter *S_o_* increased with increasing molecular weight, the conformational order parameter was found to be *S_c_* = 0.59 for MEH-PPV, independent of the chain length, see Figure 4. This further validates the previous finding on the dependence of the order parameter on the number of tetrahedral defects (number of chain segments) [60], because the percentage of tetrahedral defects (1–11%) was independent of*M* as deduced from absorption and NMR spectra [64]. These results demonstrate that the increasing number of defects for larger polymer weights inherently limits the polarization anisotropy of the polymer solute. For applications in colored LC displays, the authors suggest to achieve further increase in the dichroic ratio by use of a stiffer macromolecular solute [64].

### 4.2. Order Parameters of MEH-PPV in Nematic/Smectic 8CB

**Figure 5:**
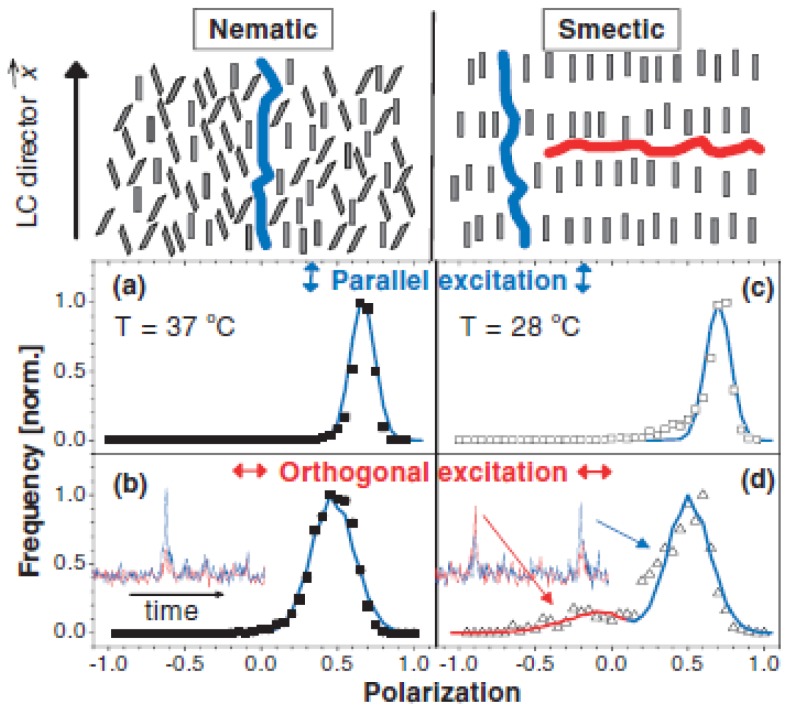
Orientations for solvation of MEH-PPV in a nematic (left) and smectic (right) LC. Top: Schematic solvation of long polymers (not scaled) is indicated by the blue and red chains. Bottom: Polarization (*P*) histograms measured with excitation polarized parallel (**a**), (**c**) and orthogonal (**b**), (**d**) to the director. The insets show a typical fluorescence burst from a single polymer molecule diffusing through the excitation volume. Positive *P* is consistent with parallel alignment to the director (blue chain). For orthogonal excitation in the smectic phase (**d**), the fluorescence transient reveals the presence of molecules with an opposite *P*. These molecules are orientated perpendicular to the director (red chain). The corresponding *P* histogram shows that a small fraction (≈10%) of polymer molecules is aligned perpendicular with a larger degree of disorder. The solid lines are theoretical fits to the histograms using an anisotropic mean-field solvation model. Image taken from [62], Copyright 2006 by The American Physical Society, with permission.

When studying the rodlike conjugated polymer MEH-PPV in smectic liquid crystal 8CB by single molecule polarization spectroscopy, Link *et al*. found most molecules would align with the LC director. However, about 10% of MEH-PPV showed a polarization perpendicular to the LC director [62]. The authors suggest these molecules to align in gaps between two smectic layers, see Figure 5. A comparison of the single-molecule polarization values in the nematic and in the smectic phase shows that for the parallel site in the smectic solvent, the MEH-PPV chain segments are aligned to a greater degree than in the nematic phase [20]. The average molecular polarization of polymer chains is smaller for those aligned perpendicular to the LC director, see Figure 5. Together with the broad distribution this reflects a greater degree of disorder. The lower polarization for the perpendicular solvation site has been assigned to various factors, such as a disordered polymer conformation, a broad orientational distribution, and/or fast molecular rotation in the y-z plane [20].

### 4.3. Diffusion Anisotropy of MEH-PPV in Nematic 5CB

To investigate the diffusion anisotropy of MEH-PPV in nematic 5CB, Link *et al*. used a two focus cross correlation technique [61]. They found a rather small diffusion anisotropy ratio, *D*_||_*=D*_⊥_ = 1.9 ± 0.3, for MEH-PPV in 5CB [20,61]. The authors compare the experimental values to predictions from theory. Models taking into account the aspect ratio and order parameter of the solute MEH-PPV [66,67] predict a much larger diffusion anisotropy. Hydrodynamic models [44,45] yield better agreement with the experiment [20]. From this the authors conclude that the diffusional anisotropy of macromolecules in anisotropic media is rather insensitive to the solute’s alignment and conformation, but is mainly dominated by the properties of the solvent [20,61]. According to Link *et al*., the experiments also shed light on the hydrodynamic boundary conditions for solute diffusion in nematic solvents. The anisotropic diffusion of rods is qualitatively different for stick and slip boundary conditions. In case of stick, the friction scales with the length of the rod in both the parallel and perpendicular directions, and the diffusion anisotropy becomes independent of the aspect ratio *e* in the limit of large *e*. In case of slip, however, the friction in the direction parallel to the rod axis is independent of the aspect ratio and the anisotropy becomes an increasing function of the length of the rod. The small value of the diffusion anisotropy for *e* = 9.2 suggests that stick boundary conditions are more appropriate for polymers in a nematic solvent [61]. This finding also agrees with the previously mentioned large hydrodynamic radii found for diffusion of small perylene diimide molecules in 5CB and in 8CB [34–36].

### 4.4. Local Structure Exploration in Electric Field Aligned LC by Use of MEH-PPV

The property of MEH-PPV to follow the LC director was used by Chang *et al*. to study the local LC structure in a parallel 5CB cell with and without an applied E-field as a function of distance from the LC substrate interface [37]. Experimental conditions were similar to those above reported for confocal microscopy with two perpendicular polarized detection channels [20,37]. The E-field studies imply that it is possible to switch the MEH-PPV alignment by controlling the direction of the anisotropic solvation potential through an E-field. Similar results had been obtained before for the smaller perylene diimide in a parallel E7 cell [32] and a twisted 5CB cell [35]. Furthermore, by scanning the laser focus across depths between 0 and 3 μm away from the LC-PVA interface, a tilt angle profile could be obtained. Above 3 μm distance to the substrate, no change in tilt angle was observed. The spatial director profile of the LC molecules with an external field is usually evaluated using Frank continuum theory, where the deformation of the LC is described by a continuous elastic theory, considering the free energy of deformation of the LC and the effect of an electric field [37]. The distance dependent tilt angles derived from polarizations of MEH-PPV qualitatively agree with these theoretical predictions [37]. This further demonstrates the power of using elongated solute molecules for the acquirement of local structure information in liquid crystals.

Besides its importance for technical applications, LC material is also found in living cells. The next section is dedicated to SM experiments studying biological LC materials and the interaction with large biomolecules.

## 5. Biological LC Materials and Their Impacts on Large Guest Molecules

Biological molecules and other microscopic entities quite often form lyotropic liquid crystals [5,68]. Dynamics of such materials and of incorporated guest molecules are therefore of particular interest for life sciences and biomimetics. Lettinga *et al*. tracked single stained *fd* viruses in an aqueous solution of rodlike *fd* viruses [38–40]. By comparing *D*_||_ and *D*_⊥_ obtained in the nematic/cholesteric and in the isotropic phase, Lettinga *et al*. conclude that *fd* is very weakly entangled in the isotropic phase. In the nematic phase, for the colloidal rod-like virus with an aspect ratio *e >* 100, diffusion anisotropies *D*_||_*=D*_⊥_ ranging from 7.5 to ≤ 20 were measured as a function of increasing order parameter [38] confirming theoretical predictions of the dependence of the diffusion anisotropy on order parameter and aspect ratio [20,65]. In the smectic phase a layer hopping motion was suggested [39,40].

**Figure 6:**
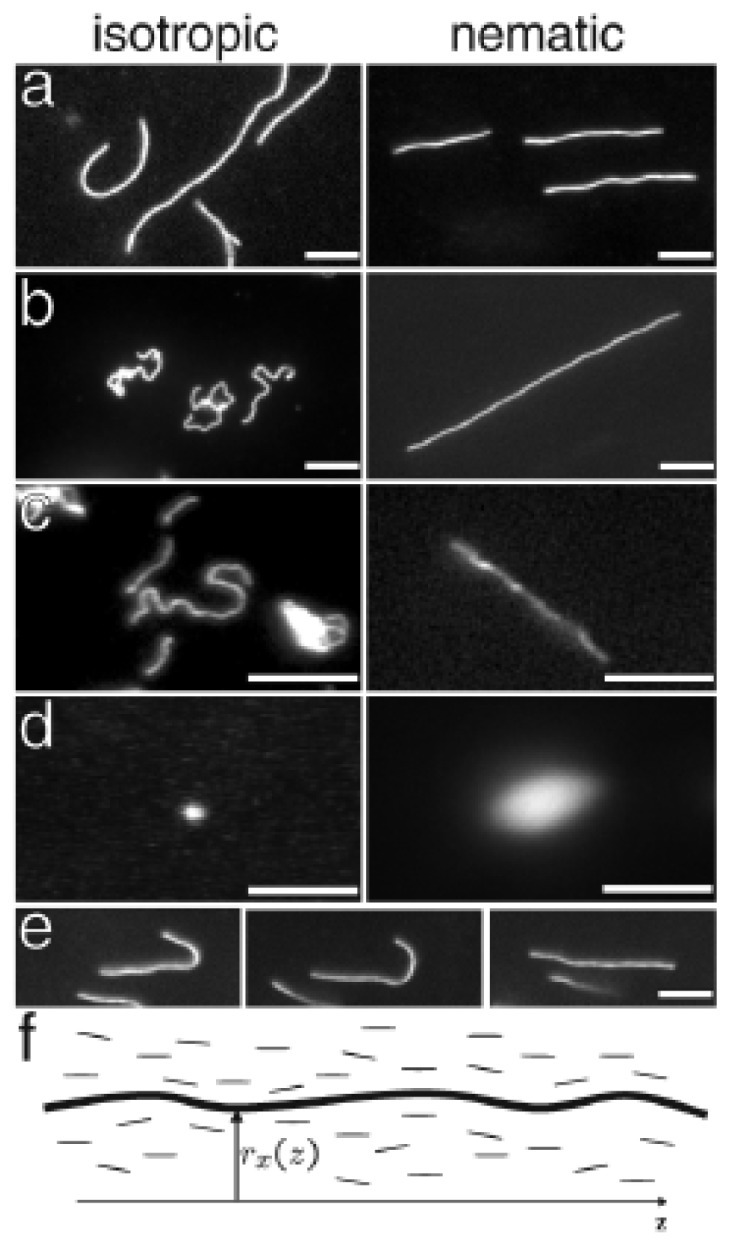
Images of fluorescently labeled biopolymers in the isotropic (left panels) and nematic (right panels) phases of the *fd* virus. (**a**)–(**d**) are, respectively, the images of actin, worm- like micelles, neurofilaments, and DNA. The polymers in an isotropic solution are confined by a thin chamber thus making the samples quasi two dimensional; (**e**) A sequence of images illustrating an actin filament escaping from a hairpin defect. The scale bar is 5 μm; (**f**) A schematic of a biopolymer in the background nematic field; the conformation of the polymer is parametrized by *R*(*z*) = {*r_x_*(*z*), *r_y_*(*z*), *z*}. The nematic director points along the *z* axis. Image and caption taken from [41], Copyright 2004 by The American Physical Society, with permission.

Dogic *et al*. studied the dynamics of three different fluorescence labelled semi flexible polymers and of wormlike micelles in the nematic and isotropic phase of an aqueous solution of the rodlike *fd* virus [41]. The persistence length of the used DNA molecules was one order of magnitude lower than that of neuro-filaments and more than two orders of magnitude lower than that of F-actin. The latter two as well as the worm like micelles could be traced in fluorescence microscopy experiments, while the DNA molecules separated from the *fd* nematic solution even at very low concentration, see Figure 6. These observations suggest that the persistence length of the polymer is important in determining its solubility in the nematic liquid crystals [41]. For actin filaments, the authors found a decrease of the order parameter *S*_actin_ with decreasing contour length. For small contour length *S*_actin_ approaches the order parameter *S*_fd_ of the smaller nematic *fd* viruses. These findings agree with the observation from conjugated polymers in thermotropic 5CB, namely that the orientational order parameter *S_o_* depends on the relative number of chain segments [60,64], constituting stiffer or more flexible chains. In the nematic phase of *fd*, F-actin, wormlike micelles, and neurofilaments, are highly elongated, having a rodlike shape in agreement with predictions from Onsager [65]. By contrast, the same filaments dissolved in an isotropic phase crumple into more compact random coils. Just above the isotropic-nematic transition, actin filaments and wormlike micelles form hairpin defects [41]. This direct visualization of individual polymer molecules yields valuable information about the behavior of polymer chains in anisotropic solvents. Since many biopolymers such as the actin filaments within the sarcomere and neurofilaments within the axon reside in an anisotropic, nematic-like environment [5], these results shed light on organization mechanisms within the cell [41].

## 6. Conclusions

Single molecule experiments on LC materials have demonstrated the validity of Onsagers prediction on the ordering of elongated molecules in anisotropic solvents [20,35,41,59,60,62,65]. By comparison of calculated hydrodynamic radii of the employed single guest molecules and the obtained diffusion coefficients with predictions from hydrodynamic models, it was shown that also single molecules lead to considerable distortions of the local LC ordering [34,35,61]. Similar to observations for spheric particles in LC materials, diffusion dynamics are best modeled by hydrodynamic models containing stick boundary conditions at the LC-guest molecule interface. The impact of stick boundary conditions on the LC material embedding guest molecules can be envisaged as an enlarged hydrodynamic radius caused by a LC shell surrounding the guest.

Furthermore, structural information on local LC alignment was obtained from SM experiments. With the use of aligning MEH-PPV molecules, it could be shown that the LC alignment in a parallel nematic cell under application of an E-field follows predictions from Frank theory [37]. Aligning perylene molecules also reported local structure related dynamics in thin frustrated smectic LC films [36], and in nematic and smectic LC cells [34].

From SMT experiments on large biopolymers in lyotropic LC material, information about the dependence of the order parameter on the contour length of the guest molecules was obtained. In the isotropic phase the previously elongated guest molecules are found to form more compact random coils [41]. These results may shed light on organization mechanisms in living cells.

The here reported results demonstrate the power of single molecule methods to resolve local structure and related dynamics in LC materials. In contrast to ensemble methods, no averaging is necessary, therefore, also rare events can be monitored, as for example the orthogonal orientation of few MEH-PPV molecules in a smectic A LC matrix [62]. Due to the high sensibility, further insight on local structure and dynamics of LC materials and on interacting guest molecules is expected from future applications of SM methods.

As can be seen from this review, up to now only few SM studies have been conducted on LC materials. The prospect of future SM investigations on the one hand is to yield still further insight into well studied liquid crystals like the above described cyanobiphenyls and *fd* viruses. For these materials ongoing theoretical research and simulations [50,69] could be compared to more sophisticated SM studies. For example, vertical structure resolution within LC films could be obtained from films on tailored reflecting substrates [52] using temperature controlled setups. One the other hand, extension of LC-approved SM methods to other LC materials and configurations, such as free standing LC films [70] and single-layer smectics [71], is featured to extend the insight on such materials. Moreover, further development of SM methods will increase the research possibilities. Recently, an improved polarization resolved method has been developed: by independently varying the polarization of excitation and emission, the so called two-dimensional polarization microscopy (2D-POLIM) is able to provide extended insight in structure and energy transfer of the investigated materials [72,73]. 2D-POLIM is applicable on SM-level dye concentrations as well as on samples containing higher fluorophore concentrations. This bears the prospect of monitoring the onset of concentration dependent features, which in particular renders it a promising tool for resolving structure and dynamics in biological LC materials.

## Figures and Tables

**Table 1:** Diffusion coefficients in μm^2^/s and diffusion anisotropy of small tracer molecules and of liquid crystal (LC) self-diffusion derived from different SM and ensemble methods at room temperature.

LC	Method	Used molecule	Sample	*D*_||_	*D*_⊥_	*D*_||_*=D*_⊥_
E7 [32]	FCS	perylene diimide	1 μm cell	18	3.0	4
E7 [33]	FCS	antraquinones	2 μm cell			5
E7 [42]	FRAP	cyanine	100 μm cell			1.2 ± 0.1
5CB [34]	SMT	perylene diimide	1 μm cell	8.47 ± 0.2	5.75 ± 0.2	1.5 ± 0.2
5CB [34]	FRAP	perylene diimide		*D̄* = 2.6 ± 0.8		
5CB [17]	NMR	LC self-diffusion	bulk	50	20	2.5
5CB [43]	FRS	methyl red	100 μm cell	20	13	1.5
8CB [34]	SMT	perylene diimide	0.5 μm cell, area 1	4.5 ± 0.2	2.9 ± 0.2	1.5 ± 0.2
8CB [34]	SMT	perylene diimide	0.5 μm cell, area 2	4.3 ± 0.2	2.8 ± 0.2	1.5 ± 0.2
8CB [36]	SMT	perylene diimide	0.2 μm film 1,2	3.8 ± 0.6	2.4 ± 0.3	1.6
8CB [35]	FCS	perylene diimide (no)	0.2 μm film 1,3	110 ± 30	0.3 ± 0.1	360
8CB [35]	FCS	perylene diimide (o)	0.2 μm film 1,3	36 ± 11	0.4 ± 0.3	90
8CB [35]	FCS	terrylene	0.2 μm film 1,3	13 ± 7	0.4 ± 0.1	30
8CB [34]	FRAP	perylene diimide		*D̄* = 3.2 ± 0.6		
8CB [17]	NMR	LC self-diffusion	bulk	25	14	1.7
8CB [43]	FRS	methyl red	100 μm cell	13	9	1.5

1 Here the LC alignment is not known, *D*_||_ and *D*_⊥_ denote the obtained fast and slow diffusion coefficients, respectively;

2 The diffusivity analysis of SMT yielded also a rare third, even faster, component *D* = 105 ± 30 μm^2^/s;

3 The diffusion components were derived from two-component 2D-fits to the FCS curves.

See text for further comments.
